# Association Between Diet, Physical Activity and Body Mass Index, Fat Mass Index and Bone Mineral Density of Soldiers of the Polish Air Cavalry Units

**DOI:** 10.3390/nu12010242

**Published:** 2020-01-17

**Authors:** Anna Anyżewska, Roman Łakomy, Tomasz Lepionka, Ewa Szarska, Ewelina Maculewicz, Andrzej Tomczak, Jerzy Bertrandt

**Affiliations:** 1 Laboratory of Food and Nutrition Hygiene, Military Institute of Hygiene and Epidemiology, Kozielska 4, 01-163 Warsaw, Poland; roman.lakomy@wihe.pl (R.Ł.); tomasz.lepionka@wihe.pl (T.L.); jerzy.bertrandt@wihe.pl (J.B.); 2 Laboratory of Physiology, Military Institute of Hygiene and Epidemiology, Kozielska 4, 01-163 Warsaw, Poland; ewa.szarska@wihe.pl (E.S.); ewelina.maculewicz@wihe.pl (E.M.); 3 Faculty of National Security, Department of Security Education, The War Studies University in Warsaw, Al. gen. Antoniego Chruściela “Montera” 103, 00-910 Warsaw, Poland; a.tomczak@akademia.mil.pl

**Keywords:** nutrition, physical activity, nutritional status, body mass index, fat mass index, bone calcification, soldiers

## Abstract

Research from recent years indicates a problem of excessive body weight among soldiers, who, due to the kind of work carried out, should possess good health and fitness levels. The aim of the study was to determine the association between diet and physical activity and the nutritional status of soldiers of the Polish Air Cavalry Units. One hundred and twenty male soldiers (aged 28 ± 5 years) completed a questionnaire (food frequency questionnaire, long-form International Physical Activity Questionnaire). Body composition was determined by bioelectrical impedance analysis, and bone calcification of the forearm was assessed by the DXA (Dual Energy X-ray Absorptiometry) densitometric method. This study confirmed the association between both the diet and physical activity and body mass index (BMI), fat mass index (FMI), and bone mineral density (BMD) expressed as T-score. Significant negative correlations were found between BMI and the frequency of consumption of cereal products, meat products and fish, and nonalcoholic beverages, between FMI and cereal products, and between BMD T-score and meat products and fish, fat, nuts, and grains, sweets and snacks, and nonalcoholic beverages. Physical activity expressed as metabolic equivalent (MET-minutes/week) negatively correlated with FMI (but not BMI) and positively correlated with the BMD T-score. This study confirmed numerous irregularities in eating behavior and in nutritional status indices; therefore, there is a need for nutritional education and further monitoring of both dietary behaviors and nutritional status of soldiers.

## 1. Introduction

Both physical activity and dietary patterns are associated with weight gain. Human nutrition is inseparable from physical activity. It is the amount of food consumed and its energy and nutritional value that correspond to the quantity of physical activity a human needs. In turn, physical activity, and more specifically, generated energy expenditure, provides the basis to determine a nutritional model and the size of energy supply required with daily food rations. It has been irrefutably demonstrated that physical activity is important in the prevention of, primarily, becoming overweight or obese, as well as noninfectious diseases [1]. Being physically active can reduce the risk of mortality, in particular from cardiovascular disease [2]. Increasing one’s energy expenditure associated with physical activity of 1000 kcal/week reduces the risk of death from all causes from 20% to 30% [3]. A greater relative risk of mortality is associated with low physical activity and matches the risks associated with hypertension, hypercholesterolemia, or obesity. When discussing the beneficial effects of regular physical activity, it should be stressed that in public health strategy, its increase is as essential as the treatment of hypertension, lipid metabolism disorders, and other nutrition-related noncommunicable diseases.

The biggest public health problem of the 21st century is an insufficient level of physical activity, resulting in the growing problem of people suffering from being overweight or obese [4]. Research from recent years indicates a problem of excessive body weight among soldiers [5,6,7,8,9]. The aero-mobile forces are part of the Polish Army land troops. These forces have the largest capabilities regarding tactical and operational maneuvers. Their ability to be deployed by helicopters and destroy the enemy makes them particularly predisposed to respond quickly. The type of operations they carry out are characterized by actions in direct contact with the enemy, often in small groups and with a risk of isolation [10,11]. Very good health and an excellent psycho-physical condition are the main factors that determine the effectiveness and reliability of the performance of special tasks in the case of soldiers serving in Air Cavalry units. Hence, assessment of the health, especially nutritional status of soldiers, is an important element of evaluation of their suitability for service. Among many measures of the nutritional status, nutritional protein-energy indicators are distinguished, i.e., body mass index (BMI) and fat mass index (FMI), as well as an indicator of mineral nutrition, bone mineral density (BMD). Moreover, proper nutritional status of soldiers is extremely important, as it significantly affects their physical fitness [12]. Disorders of nutritional status occurring among soldiers, such as being underweight, overweight, or obese, which result mainly from improper diet, not only impair well-being but are social and health problems associated with soldiers’ having a limited potential to perform tasks and duties and can lead to earlier elimination from the service. The lack of consistency has been seen across studies assessing associations of obesity with diet, and underlying mechanisms of weight gain are still being elucidated [13,14,15,16,17,18]. Weight can be stored as fat mass or lean mass. Therefore, it is crucial to consider associations between diet and both body mass and fat mass. In Poland, the new Pyramid of Healthy Nutrition and Lifestyle [19], developed by the National Food and Nutrition Institute, is used to promote healthy nutrition for the general population and can be also used in military units.

Studies on diet and physical activity of soldiers serving in the Polish Army are few and fragmented. There is a lack of research combining aspects of soldiers’ lifestyles with their nutritional status. Therefore, the aim of the undertaken research was to determine the association between diet and physical activity and selected nutritional status indicators BMI, FMI, and BMD of soldiers of the Polish Air Cavalry Units.

## 2. Materials and Methods

The study was carried out in the autumn, with the participation of 120 soldiers (all members of the unit) serving in one of the Air Cavalry units of the Polish Army. All subjects gave their informed consent for inclusion before they participated in the study. The study was conducted in accordance with the Declaration of Helsinki, and the protocol was approved by the Ethics Committee of 1/XXI/2016. Assessment of dietary habits and physical activity of soldiers, as well as nutritional status, including bone calcification, was carried out as part of the research.

### 2.1. Food Habits and Food Consumption Frequency

A questionnaire consisting of two parts was used to assess dietary habits. The first part comprised questions related to the regularity and location of meals that were eaten during the day, snacking on sweets, fast-food products, and the drinking of sweetened sodas during the day. After being validated for the Polish population, a 61-item food frequency questionnaire (FFQ) [20] formed the second part of the questionnaire. The FFQ was modified for our own research by adding two additional categories of answers regarding frequency of consumption, with the potential to choose between quarterly or less often and once a week. Participants were asked how often, on average, they had consumed food products and beverages included in the FFQ. The study identifies eight food products groups, including:

1. Fruits, vegetables, seeds of legumes and potatoes: fruits together—all types; stone fruits; kiwi fruit and citrus fruits; other tropical fruits; bananas; apples and pears; avocado; olives; dried fruits; sweet fruit preserves and candied fruits; vegetables—all types; crucifers; yellow-orange vegetables; green leafy vegetables; tomatoes; vegetables including fresh cucumbers, squash, zucchini, pumpkin, and eggplant; root vegetables and others; fresh seeds of legumes and canned ones; dry seeds of legumes; potatoes in various forms.
2. Cereal products: wholemeal or with grains, so-called dark bread; refined bread, so-called white bread; unrefined groats coarse; refined cereal grain; ready-to-eat breakfast cereal products.
3. Dairy products and eggs: milk and milk drinks; sweetened milk drinks; cottage cheese; flavored cottage cheese; cheese; eggs and egg dishes.
4. Meat products and fish: sausages—different types; high-quality cold cuts; sausage products and offal; red meat; poultry and rabbit; wild game meat; lean fish; oily fish.
5. Fats, nuts, and grains: oil—all kinds; butter—all types; margarine—all types; cream, sweet or sour cream for food or beverages; other animal fats; mayonnaise and dressings, i.e., salad dressings—all types; nuts; grains.
6. Sweets and snacks: sugar to sweeten beverages; honey to sweeten food and beverages; chocolate, chocolate candies, and candy bars; non-chocolate candies; biscuits and cakes; ice cream and pudding; salty snacks.
7. Soft drinks: fruit juices and fruit nectars; vegetable juices and vegetable–fruit ones; energy drinks; sweetened sodas such as Fanta, Coca-Cola, Mirinda, Sprite.
8. Alcoholic beverages: beer; wine and drinks; vodka and spirits.

Respondents could select one of eight categories regarding the consumption frequency: 1—never or almost never, 2—once a quarter or less often, 3—once a month or less often, 4—a few times a month, 5—once a week, 6—several times a week, 7—every day, 8—several times a day. In order to conduct further analysis, the data reported in the FFQ were transformed into daily frequencies. Daily frequencies of each food were calculated using the following values for reported frequencies: never or almost never—0.003 (1/365), once a quarter or less often—0.01 (4/365), once a month or less often—0.03 (1/30), a few times a month—0.08 (2.5/30), once a week—0.14 (1/7), several times a week—0.57 (4/7), every day—1, several times a day—2. Converted for each person and product units of time/day were also summed within food groups. Food frequency consumption covering the past 12 months was collected. Soldiers were living at home and provided their own food.

### 2.2. Physical Activity

Physical activity was assessed using a long-form International Physical Activity Questionnaire (IPAQ) [21] which consisted of 27 questions about physical activity, including:

- job-related physical activity,
- transportation physical activity,
- housework, house maintenance, and caring for family,
- recreation, sport, and leisure-time physical activity,
- time spent sitting.

Physical activity was expressed as metabolic equivalent (MET-minutes/week unit), as a product of days in a week (in the last 7) in which particular activity was undertaken, its duration, and the factor assigned to each activity (depending on its intensity), specified in the scoring form. Total physical activity and physical activity within particular categories, as well as physical activity associated with walking and moderate and intense physical activity, were calculated. The percentage of people with a high, moderate, or low level of physical activity, in accordance with obligatory criteria, was also determined [22]:

1. High level of physical activity—any one of the following criteria:
  - vigorous-intensity activity on at least 3 days and accumulating at least 1500 MET-minutes/week OR
  - 7 or more days of any combination of walking, moderate-intensity, or vigorous-intensity activities achieving a minimum of at least 3000 MET-minutes/week.
2. Moderate level of physical activity—any one of the following criteria:
  - 3 or more days of vigorous activity of at least 20 min per day OR
  - 5 or more days of moderate-intensity activity or walking of at least 30 min per day OR
  - 5 or more days of any combination of walking, moderate-intensity, or vigorous-intensity activities achieving a minimum of at least 600 MET-min/week.
3. Low level of physical activity—those individuals who do not meet the criteria for a high or moderate level of physical activity are considered low/inactive.

### 2.3. Nutritional Status

#### 2.3.1. Anthropometry, Body Composition

Height, body mass, and body composition measures were taken when participants were wearing light clothing and no shoes. Height (cm) was measured with a portable stadiometer with the head in the horizontal Frankfurt plane and recorded with a range of 0–2.10 m and with a precision of 0.1 cm (TANITA HR-001, Tanita Corporation, Japan). The body mass and the multi-frequency bioelectrical impedance measurements were performed with the TANITA MC-780 analyzer (Tanita Corporation, Japan). The measure consisted in a stand-alone unit which the subject had to step on barefoot (standard mode). Once body mass (kg) had been assessed by the electric scale with a range of 0–270 kg and with the precision of 0.1 kg, the subject had to take grips in both hands (alongside his body) during the impedance measure (hand-to-foot method; 8-polar). Arms were not touching sides and inner thighs were not touching each other during measurement. The bioelectrical impedance analysis was realized at least three hours after a meal (at midday) without any intensive exercise before measure or on the day before. All measurements were performed according to the procedure specified in the instruction manual and with metal objects (e.g., jewelry, keys) removed. Fat mass (kg), along with its percentage (%), was determined. Indexes for body mass, fat mass, and fat-free mass were calculated; i.e., body mass index (BMI) = body weight/height^2^ (kg/m^2^), fat mass index (FMI) = fat mass/height^2^ (kg/m^2^), and fat-free mass index (FFMI) = fat-free mass/height^2^ (kg/m^2^). Assessment of the BMI values was made in accordance with the criteria set out by the World Health Organization (WHO) [23]. The scale of FMI classification developed by Kelly et al. was accepted [24], adopting FMI values between 3 and 6 as a normal fat mass, FMI < 3 as fat deficit, FMI > 6 as excess fat.

#### 2.3.2. Bone Calcification

Bone calcification was assessed by the DXA (Dual Energy X-ray Absorptiometry) densitometric method, measuring the BMD of forearm bone of the nondominant hand using the EXA 3000 apparatus (OsteoSys, Korea; software version: 2.02.11a). The results were interpreted based on the BMD T-score value in accordance with the WHO standards [25], i.e., standard: BMD T-score > −1.0; osteopenia: −2.5 < BMD T-score ≤ −1.0; osteoporosis: BMD T-score ≤ −2.5.

### 2.4. Statistical Analysis

Statistical analysis was performed by the STATISTICA ver. 13 software. The compatibility of variables distribution with normal distribution was assessed by the Shapiro–Wilk test, accepting the significance level of α = 0.05. Data were described by an average value with standard deviation, as well as by medians and interquartile ranges in the case of noncompliance with a normal distribution. The rho-Spearman’s correlation (due to noncompliance with a normal distribution) was used to assess relationships between diet, physical activity, and BMI, FMI, and BMD T-score. In order to verify relationships between snacking on sweets, fast-food products or sweetened sodas, and the consumption of different products and nutritional status, the student’s T-test or the Mann–Whitney U test was used, depending on the compatibility of variables distribution with normal distribution. The Mann–Whitney U test was also used to compare food frequency consumption between soldiers with adequate and inadequate nutritional status indices (BMI, FMI, and BMD T-score). A significance level of α = 0.05 was assumed in all analyses. The level of statistical trends for *p* is in the range of 0.05 to 0.1, and this was assumed to be due to the low number of selected subgroups, depending on snacking on selected products, as well as in the case of appropriate/unsatisfactory nutritional status groups.

## 3. Results

A total of 120 men aged from 20 to 41, with an average of 28 ± 5 years, took part in this study. The average length of service was 5 ± 5 years. A quarter of the examined subjects lived in a city of over 100 thousand inhabitants, one-third lived in a city of up to 100 thousand inhabitants, and the rest lived in the countryside. The physical intensity of work was assessed as moderate by almost half of the subjects (49%). According to 37% of soldiers, their work was hard, and for others was light or performed while sitting.

### 3.1. Food Habits and Food Consumption Frequency

In the examined unit, only 69% of the soldiers ate breakfast every day, and the majority of them (58%) only ate their first meal at work (Table 1). Lunch was eaten every day by half the soldiers (51%). Almost everybody (90%) ate dinner every day, most often at home (75%). An afternoon snack was eaten every day only by one-fifth of the soldiers and supper by nearly three-quarters of the subjects. The above-mentioned abnormalities in the frequency of eating meals indicate the general poor eating habits.

Fruits were eaten every day by only 30% of soldiers, including 8% of them who ate these products several times a day (Table 2). Half of the subjects ate fruits several times a week, and the other 20% of soldiers ate these products once a week or less frequently. Bananas were the most common fruit consumed (59% of soldiers ate bananas more than once a week), then apples and pears (53% of soldiers ate apples and pears more than once a week), while avocado and olives were the least-often consumed fruits (4% of soldiers ate avocado, and 8% ate olives more than once a week). Vegetables were eaten every day by only one-third of the soldiers (33%), and only 4% of those examined ate vegetables several times a day, 44% of subjects ate vegetables several times a week, and the remaining 23% ate them once a week or less frequently. Tomatoes were the vegetable that was consumed most often (59% of soldiers ate tomatoes more than once a week), and green leafy vegetables were the least-often consumed vegetables (25% of soldiers ate green leafy vegetables more than once a week). Wholemeal bread or bread with grains was eaten daily by less than one-third of the soldiers examined, and unrefined groats coarse was consumed by only 9%. Dairy products were consumed by most of the soldiers less than daily. Sausages and high-quality cold cuts were the most popular meat products, while fish were eaten by approximately three-quarters of the soldiers examined less than once a week. Sweets (47%) and sweetened noncarbonated drinks (42%) were the most often consumed between-meal snacks, but fast-food products were not as popular (17% of indications).

### 3.2. Physical Activity

The level of physical activity, based on responses in the IPAQ, was classified as high for 90% of the subjects. The remaining 10% was characterized by a moderate level of physical activity. The calculated total physical activity amounted to 15,810 MET-minutes per week on average (median: 12,087; interquartile range: 8764–22,341 MET-minutes per week), of which the biggest part was physical activity associated with work (39%), and the smallest was physical activity associated with transportation (15%) (Table 3). Housework, general house maintenance, and caring for family accounted for 24%, and recreation, sport, and leisure-time physical activity was 21% of the total amount of physical activity. Almost half of the physical activity of the subjects was characterized by moderate intensity (49%), intensive physical activity was close to one-third (32%) of the total value, and walking counted for the remaining 19%. Time spent in the sitting position was very diverse and ranged from 1 to 14 h a day, both on weekdays (median: 3; interquartile range: 2–4) and on weekends (median: 4; interquartile range: 2–5).

### 3.3. Nutritional Status

The group of soldiers examined was characterized by a large variety of assessed anthropometric indicators. The body weight of examined soldiers ranged from 59.4 to 116.4 kg, while body height ranged from 167.0 to 198.0 cm (Table 4). The BMI value ranged from 18.4 to 32.8 kg/m^2^, and the FMI value ranged from 0.9 to 9.7 kg/m^2^, while the percentage of fat content ranged from 4.9% to 29.7%, an average of 17.5% ± 4.5% Not all the soldiers examined revealed appropriate nutritional status in relation to body size (BMI), obesity index (FMI), and bone calcification (BMD T-score). Normal BMI was found in 41% of soldiers, and the adequate FMI was found in 68% of the subjects. More than half of those examined (58%) were classified into a group where the BMI was above the norm, i.e., > 25 kg/m^2^, while an excessive amount of fat tissue, as interpreted by the FMI, was found among only 19% of the soldiers. Sufficient bone calcification was found in almost all subjects (92%).

### 3.4. Association Between Diet, Physical Activity, and the Body Mass Index, Fat Mass Index, and Bone Mineral Density

Dietary habits (Table 5) and the level of physical activity (Table 6) correlated with BMI, FMI, and bone calcification. Eight statistically significant negative correlations between the consumption frequency of five out of the eight listed groups of products were noticed; between cereal products and BMI (rho = −0.22, *p* = 0.031) and FMI (rho = −0.25, *p* = 0.012), between meat products and fish and BMI (rho = −0.23, *p* = 0.021) and BMD T-score (rho = −0.23, *p* = 0.024), between fat, nuts, and grains and BMD T-score (rho = −0.22, *p* = 0.030), between sweets and snacks and BMD T-score (rho = −0.27, *p* = 0.007), and between nonalcoholic beverages and BMI (rho = −0.31, *p* = 0.002) and BMD T-score (rho = −0.32, *p* = 0.001). Out of the 61 products selected, statistically significant correlations between BMI and eight products (refined bread, sausages, high-quality cold cuts, butter, margarine, fruit juices and nectars, sweetened sodas, vodka and spirits) and between FMI and three products (apples and pears, sausages, vodka and spirits) were found. 

The BMD T-score value positively correlated with the frequency of tropical fruits, refined cereal grain, and eggs consumption. A negative correlation was found for the frequency of refined bread, sausages, high-quality cold cuts, butter, mayonnaise, sugar to sweeten beverages, biscuits and cakes, salty snacks, juices and fruit nectar consumption, and for sweetened sodas such as Fanta or Coca-Cola. In addition, it has been shown that the mean value of bone mineralization (BMD) in groups of subjects eating fast-food products or drinking sweetened sodas was significantly lower than among those who did not eat these products; this amounted to, for fast-food, 0.606 vs. 0.561 g/cm^2^ (BMD T-score: 0.358 vs. −0.400, *p* = 0.007); and for sweetened sodas, 0.618 vs. 0.581 g/cm^2^ (BMD T-score: 0.542 vs. −0.075, *p* = 0.001), respectively. In the case of snacking on sweets, the difference was only at the level of statistical trends (BMD: 0.610 vs. 0.590 g/cm^2^; BMD T-score: 0.418 vs. 0.084, *p* = 0.082). Eating any of these products did not change FMI values (*p* > 0.1). 

Additional analyses also confirmed associations between foods eaten and BMI, FMI, and BMD T-score; a comparison of food frequency consumption between soldiers with adequate and inadequate nutritional status indicators is presented in Supplementary Materials (Table S1).

There were no correlations between BMI and physical activity assessments, while a negative correlation with FMI (rho = −0.22, *p* = 0.036) for weekly physical activity expressed in MET-minutes/week was found. Positive correlations were found between BMD T-score and physical activity expressed in MET-minutes per week in total and two of its components, i.e., housework, house maintenance, and caring for family (rho = 0.22, *p* = 0.040); and recreation, sport, and physical activity in leisure time (rho = 0.25, *p* = 0.019), as well as moderate physical activity (rho = 0.28, *p* = 0.008) and intense physical activity (rho = 0.21, *p* = 0.046). Taking into consideration the level of statistical trends, it was shown that subjects who spent more time sitting, both on weekdays and weekends, are characterized by higher values of the FMI than subjects who spend less time sitting. A relationship between time spent sitting, neither during the week nor at the weekend, and bone calcification was not found.

## 4. Discussion

We investigated abnormalities in soldiers’ diets and nutritional status, as well as the associations between soldiers’ dietary behaviors and BMI, FMI and BMD T-score. The results indicated poor diet among Polish soldiers: 31% of the participants did not eat breakfast, 49% did not eat lunch, and about 70% of them consumed fruits and vegetables less often than once a day. As categorized by fat mass index cut-points, 19% of soldiers had excess fat and 13% a fat deficit and from the measure of bone mineral density, and 8% of soldiers were classified as osteopenic.

The model of a soldier’s diet was not compatible with the current recommendations of the National Food and Nutrition Institute [19]. Plant-origin foods (fruit and vegetables) are situated at the base of the healthy eating pyramid [19], and due to their nutritional value, these products should be consumed in high proportions and frequently. Meanwhile, these products were consumed by most of the soldiers less than daily. Similar nutritional deficits, i.e., insufficient frequency of fruits and vegetables and milk and dairy products consumption by Polish soldiers, have been shown in previous years by Hyżyk et al. [26]. Studies carried out in the United States [27] showed that during several months of deployment, the quality of diets that soldiers of special operation forces were fed deteriorates, in particular, the consumption of fruits, vegetables, and dairy products was reduced.

In accordance with The Physical Culture Regulations for the Armed Forces of the Republic of Poland, soldiers of special units must have six hours of physical education per week, and in the case of other soldiers, the requirement is four hours. In this study, a high level of physical activity was found in almost all soldiers, while among the citizens of Europe it ranges from 23% (Sweden) to 44% (The Netherlands), and in Poland, it amounts to 33% [28,29]. However, Tomczak [30] pointed out the diversity of levels of physical activity among Polish soldiers, showing significant differences in physical activity of soldiers from special units and soldiers working in the military administration units. In this study, no correlation between physical activity and BMI was observed; however, FMI negatively correlated with total physical activity (MET-minutes/week) and the intensive physical activity. Correa-Rodríguez et al. [31] showed that moderate-to-vigorous physical activity in young adults was significantly associated with BMI, while there was no correlation with fat mass.

In recent years an increase in the BMI value among soldiers is clearly visible [5,6,7,8,9], similarly as in the whole population. BMI is simple to calculate, and therefore is often used in clinical assessments around the world. However, many analyses indicate the absence of strict correlation with the percentage of body fat, and therefore excessive body weight resulting from excessive body fatness [32,33]. Relatively poor correlation between these indicators has also been found among participants of the Health and Nutrition Examination Survey (NHANES) III, revealing quite wide ranges of the percentage of body fat both among men (14%–35%) and women (26%–43%) subjects who had a BMI of 25 kg/m^2^ [34]. Moreover, due to difficulties in BMI interpretation in adults with increased physical activity, it seems more reasonable to assess the appropriateness of nutritional status on the basis of an analysis of the FMI value, as this indicator assesses the degree of fatness, and not the entire body weight, which in the case of soldiers is usually greater due to extensive muscle mass, which can increase the BMI value [35,36]. In our study, the average BMI among soldiers was above reference values, while the average FMI was adequate. Researchers often suggest caution when interpreting BMI in adults with increased physical activity [35,37,38]. On the other hand, Grier et al. [39] showed that age-adjusted BMI values of U.S. soldiers are effective in estimating body fat and determining the occurrence of people who are overweight and obese. 

The values of BMI obtained in this study were similar to the results of tests carried out with the participation of 99 soldiers from another aero-mobile forces unit [40]. The average BMI value, just like among soldiers from the U.S.A. [7], was outside the range of reference values (18.5–24.9 kg/m^2^), and the percentage of subjects revealing excessive body weight, assessed based on the BMI, was slightly lower in this study compared to soldiers from the United States (58% vs. 64%) [7]. Soldiers from the U.S.A. were characterized by a wider range of BMI values (19.5–40.7 vs. 18.4–32.8 kg/m^2^). The average fat content of the soldiers examined remained at the same level as among soldiers from the U.S.A. in total (17.5%) [39], as well as among special operations personnel (18.0%) [41].

A systematic review of cohort studies provided moderate-quality evidence for an inverse association between vegetable intake and weight-related outcomes in adults [42]. Vegetables and fruit have similar properties; however, the effect on weight might differ [43]. Fruit contains a larger amount of simple sugars and contains less water and fiber than nonstarchy vegetables. Epidemiological studies have shown that fruit has an antiobesity effect; however, there are also probable mechanisms for the pro-obesity effect of fruit, such as that fruit increases simple sugars intake [44]. In this study, negative correlations between the frequency of selected fruits consumption and BMI or FMI were found, while there were no significant correlations between BMI or FMI and vegetable consumption. BMI positively correlated with the frequency of vodka and spirit consumption, while FMI was positively correlated with the frequency of beer and vodka and spirits consumption. Van Eekelen et al. [45] showed that consumption of each extra daily alcoholic beverage was associated with increased liver fat in middle-aged men and women. Associations between the frequency of consumption of other products (i.e., cereal products) and BMI or FMI among soldiers seem to be unclear. Further research is needed to clarify the possible influence of diet on energy-protein nutritional status markers.

Another measure of nutritional status that is equally important in case of soldiers is bone mineral density (calcification), which, if it is too low, may lead to osteopenia and later on to osteoporosis, increasing the risk of fractures. Studies do not show a close relationship between osteoporosis and the intake of other nutrients besides calcium and vitamin D, although it is believed that reduction in sodium intake and increased potassium intake may be beneficial, as well as consumption of vegetables and fruits [46]. In addition, researchers enumerate low intake of vitamins and high consumption of alcohol, caffeine, and sweetened sodas as nutritional risk factors for osteoporosis [47]. Other factors include genetic, hormonal, and environmental factors and lifestyle, and in particular, physical activity from an early age [48,49]. Almost all subjects (92%) revealed a proper BMD (BMD T-score above −1); no changes typical of osteoporosis were found among the soldiers examined. Similar results were obtained in studies carried out in other units of land forces in Poland, where adequate bone calcification was found in 75% of soldiers aged up to 30, among 90% of soldiers aged over 30 (Airborne Battalion) [40], among 77% of soldiers returning from a mission in Afghanistan, aged up to 30, and among 87% of soldiers aged over 30 [50].

Like other studies [51,52], this study has demonstrated positive correlations between physical activity, in particular, total, moderate, and that associated with sports or recreation, and BMD expressed as T-score indicator. Significant positive correlations between calcaneus broadband ultrasound attenuation and moderate and high levels of physical activity were also observed in young Spanish adults [53]. Alghadir et al. [52] showed the negative impact of frequent consumption of sweetened sodas on BMD. Authors underlined that these dependencies are particularly important in cases of low milk consumption. However, in this study, there was no relationship between the consumption of dairy products and the BMD T-score values, probably because almost all subjects had appropriate bone calcification, i.e., BMD T-score above −1. Tucker et al. [54] have shown that there is no close relationship between the entire group of sweetened soda products in total and BMD, whereas relationships occur in the case of cola drinks, which can most likely be explained by a large content of phosphoric acid. Excessive intake of phosphorus as a risk factor for bone health has also been shown in other studies by Calvo and Tucker [55], pointing to the possible mechanism, among others, of an imbalance of calcium phosphate in the body. Drinking sweetened sodas and eating fast-food products by soldiers also affected bone density. Similar adverse effects in both mineral and protein-energy nutritional status, arising from the physical load and replacing meals with fast-food products, has been also found among students from the Main School of Fire Service [56].

## 5. Strengths and Limitations

The present study has several strengths. First, there are limited numbers of studies among soldiers that concurrently assess the nutritional status (anthropometry, body composition, and bone calcification) and nutrition and physical activity. Second, we used validated questionnaires: FFQ (including eight food products groups and 61 items) [20] and long-form IPAQ [21], and we focused on three nutritional status indicators: indicators of protein-energy status describing body size (BMI) and obesity (FMI) and indicators of mineral nutritional status (BMD T-score) that seem to be important elements of the evaluation of soldier’s health and suitability for military service. Third, we found abnormalities in nutritional status and numerous nutritional inadequacies, as well as the association between both foods eaten and physical activity and the BMI, FMI, and BMD T-score. Fourth, we showed that it is necessary to perform educational activities in the field of health promotion of soldiers, with a focus on nutritional prevention of nutrition-related noncommunicable diseases, as well as to motivate soldiers to respect the basic principles of proper nutrition.

The main limitation is the relatively small group (120 male soldiers). However, all participants served in the same Air Cavalry unit; therefore, it was representative of a sample of this type of military forces. The other limitation of this study is the assessment of the BMD on the forearm. We agree that BMD measured on the hip, spine, or total body are more highly recommended and more precise. However, there are other studies that use a forearm bone BMD [40,50,56,57] and the forearm is still one of the standard locations for DXA measurements [58]. Moreover, we conducted our study in the military unit; thus, we had the possibility to use only small-sized devices. There is one more limitation that should be kept in the mind when considering results of the present study. Since no ideal method to collect dietary data exist, FFQ is widely used in nutritional epidemiology. In our study, we focused only on the frequency of the consumption of food groups, and for this reason, some of the correlations (i.e., with cereals products) are difficult to interpret. Thus, future studies should also include portions of consumed foods to examine total energy intake and energy contributions from carbohydrates, protein, and fat, as well as to determine associations between nutritional status such as BMI and FMI not only with frequencies of food groups but also with total energy intakes.

## 6. Conclusions

The studies carried out have confirmed the associations between both foods eaten and physical activity and selected measures of soldiers’ nutritional status, i.e., BMI, FMI, and BMD T-score. Obtained results are particularly worrying due to the self-served style of soldiers’ diets, as a result of poor eating habits. The reported abnormalities in nutritional status, as well as abnormalities in nutrition, indicate a need for further monitoring of this group of soldiers, not only in terms of the accuracy of nutritional status indices, their diet, and physical activity but also wider-understood health behavior.

It is necessary to perform educational activities in the field of health promotion for this group of people, with a focus on nutritional prevention of nutrition-related noncommunicable diseases, as well as to motivate soldiers to respect the basic principles of proper nutrition.

## Figures and Tables

**Table 1:** Frequency and location of eating meals by soldiers (%).

Percentage of Answers (%)	Breakfast	Lunch	Dinner	Afternoon Snack	Supper
Every day	69	51	90	20	73
Not every day	27	34	7	36	25
Never	4	15	3	44	2
Work	58	98	25	4	4
Home	42	2	75	96	96

**Table 2:** Food Consumption Frequency.

Food Groups	Percentage of Consumption Frequency *							
	1	2	3	4	5	6	7	8
Fruits, vegetables, seeds of legumes, and potatoes								
Fruits together—all types	1	0	3	13	3	50	22	8
Stone fruits	3	4	20	25	24	19	5	0
Kiwi fruit and citrus	0	6	15	27	25	22	5	0
Other tropical fruits	3	19	28	29	10	8	3	0
Bananas	0	0	2	15	24	41	17	1
Apples and pears	0	1	3	18	25	37	15	1
Avocado	37	32	18	6	3	3	1	0
Olives	35	20	19	14	4	6	2	0
Dried fruits	14	27	25	18	3	10	3	0
Sweet fruit preserves and candied fruits	10	15	32	24	8	10	1	0
Vegetables—all types	1	1	3	11	9	44	28	3
Crucifers	0	4	7	32	25	27	5	0
Yellow–orange vegetables	0	2	3	24	31	32	7	1
Green leafy vegetables	3	7	13	29	23	22	2	1
Tomatoes	0	1	3	15	22	40	17	2
Vegetables: fresh cucumbers, squash, zucchini, pumpkin, eggplant	1	2	13	20	15	36	12	1
Root vegetables and others	0	3	9	23	20	34	11	0
Fresh seeds of legumes and canned ones	6	15	18	35	13	11	2	0
Dry seeds of legumes	7	19	28	29	13	3	1	0
Potatoes in various forms	0	3	7	18	12	45	15	0
Cereal products								
Wholemeal or with grains, so-called dark bread	3	4	5	12	14	35	24	3
Refined bread, so-called white bread	3	5	8	7	14	33	25	5
Unrefined groats coarse	3	7	13	25	26	17	8	1
Refined cereal grain	3	7	13	20	11	38	8	0
Ready-to-eat breakfast cereal products	7	14	16	22	13	16	12	0
Dairy products and eggs								
Milk and milk drinks	0	2	6	16	11	38	24	3
Sweetened milk drinks	6	6	10	22	21	27	8	0
Cottage cheese	0	2	8	21	22	38	6	3
Flavored cottage cheese	13	15	24	22	13	12	1	0
Cheese	3	4	12	18	12	40	10	1
Eggs and egg dishes	0	1	1	17	21	41	18	1
Meat products and fish								
Sausages, different types	2	3	7	17	12	41	13	5
High-quality cold cuts	2	1	2	13	17	46	17	2
Sausage products and offal	7	8	22	25	23	15	0	0
Red meat	0	7	12	19	23	36	3	0
Poultry and rabbit	2	1	4	15	14	51	12	1
Wild game meat	35	35	15	9	3	3	0	0
Lean fish	2	14	23	29	21	8	2	1
Oily fish	5	16	24	31	14	8	1	1
Fats, nuts, and grains								
Oil, all kinds	4	4	9	14	20	38	11	0
Butter, all types	3	4	4	13	8	34	29	5
Margarine, all types	17	17	14	19	9	13	8	3
Cream, sweet or sour cream, for food or beverages	8	13	20	22	14	20	3	0
Other animal fats	22	22	25	18	7	5	1	0
Mayonnaise and dressings, i.e., salad dressings—all types	5	11	24	29	13	13	4	1
Nuts	2	11	15	28	23	15	4	2
Grains	8	24	29	18	9	9	3	0
Sweets and snacks								
Sugar to sweeten beverages	24	5	7	3	6	21	25	9
Honey to sweeten food and beverages	16	21	16	20	8	11	5	3
Chocolate, chocolate candies, and candy bars	2	9	14	20	24	26	3	2
Non-chocolate candies	8	14	32	29	9	7	0	1
Biscuits and cakes	3	13	19	35	14	13	3	0
Ice cream and pudding	7	33	35	18	6	1	0	0
Salty snacks	7	21	29	20	14	8	0	1
Soft drinks								
Fruit juices and fruit nectars	3	6	3	20	13	39	13	3
Vegetable juices and vegetable-fruit ones	8	12	13	32	19	13	3	0
Energy drinks	15	13	14	25	12	18	3	0
Sweetened sodas such as Fanta, Coca-Cola, Mirinda, Sprite	12	9	15	17	11	32	4	0
Alcoholic beverages								
Beer	6	4	24	17	29	15	5	0
Wine and drinks	20	16	26	17	18	3	0	0
Vodka and spirits	10	18	36	19	14	3	0	0

* Consumption frequency categories: 1—never or almost never; 2—once a quarter or less often; 3—once a month or less often; 4—a few times a month; 5—once a week; 6—several times a week; 7—every day; 8—several times a day.

**Table 3:** Physical activity specified by soldiers in the long-form International Physical Activity Questionnaire (IPAQ).

Characteristics	X ± SD	Me	IRQ
**Physical activity (MET-minutes/week):**			
total	15,810 ± 10,502	12,087	8764–22,341
job-related physical activity	6184 ± 5537	5543	1413–9003
movement	2446 ± 2903	1386	396–303
housework, house maintenance, caring for family	3822 ± 4575	2220	960–4835
recreation, sport, and leisure-time physical activity	3357 ± 2999	2457	1106–5070
walking	3047 ± 2966	2079	693–4505
moderate	7751 ± 7260	6150	2454–9346
intensive	5012 ± 4835	3960	1584–7128
**Time spent sitting (h/day):**			
weekdays	3.7 ± 2.8	3.0	2.0–4.0
weekend	4.4 ± 2.8	4.0	2.0–5.0

X—average; SD—standard deviation; Me—median; IRQ—interquartile range.

**Table 4:** Anthropometry and selected nutritional status indices.

Characteristics	X ± SD	Me
Height (cm)	179.2 ± 6.2	179
Weight (kg)	82.1 ± 9.5	81.8
BMI (kg/m^2^)	25.6 ± 2.6	25.5
FMI (kg/m^2^)	4.6 ± 1.6	4.4
FFMI (kg/m^2^)	20.9 ± 1.4	21
**Percentage of Classifications**		
BMI (kg/m^2^)	Underweight < 18.5	1
	18.5 ≤ Norm < 25.0	41
	25.0 ≤ Overweight < 30.0	52
	Obesity > 30.0	6
FMI (kg/m^2^)	Fat deficit < 3	13
	3 ≤ Normal fat ≤ 6	68
	Excess fat > 6	19
BMD T-score	Osteoporosis ≤ −2.5	0
	−1.0 ≥ Osteopenia > −2.5	8
	Standard > −1.0	92

X—average; SD—standard deviation; Me—median; BMI—body mass index; FMI—fat mass index; FFMI—fat-free mass index; BMD T-score—bone mineral density expressed in relation to a reference population in standard deviation units.

**Table 5:** Relationship between the foods eaten (FFQ) and body mass index (BMI), fat mass index (FMI), and bone mineral density expressed as T-score.

Groups of foods	BMI		FMI		BMD T-Score	
	rho	*p*	rho	*p*	rho	*p*
**Food groups**						
Fruits, vegetables, seeds of legumes, and potatoes	−0.03	0.739	−0.11	0.291	0.04	0.668
Cereal products	−0.22	0.031 **	−0.25	0.012 **	0.07	0.478
Dairy products and eggs	−0.13	0.210	−0.16	0.114	0.06	0.573
Meat products and fish	−0.23	0.021 **	−0.10	0.325	−0.23	0.024 **
Fats, nuts, and grains	−0.15	0.134	−0.11	0.297	−0.22	0.030 **
Sweets and snacks	−0.13	0.205	−0.07	0.467	−0.27	0.007 **
Non-alcoholic beverages	−0.31	0.002 **	−0.19	0.052 *	−0.32	0.001 **
Alcoholic beverages	0.10	0.311	0.19	0.055 *	−0.10	0.348
**Selected foods**						
Fruits, vegetables, seeds of legumes, and potatoes						
Fruits together—all types	−0.01	0.932	−0.09	0.377	0.07	0.490
Stone fruits	0.10	0.345	−0.02	0.839	0.14	0.171
Kiwi fruit and citrus	0.00	0.989	−0.09	0.384	0.06	0.557
Other tropical fruits	0.08	0.459	−0.03	0.765	0.23	0.024 **
Bananas	−0.18	0.078 *	−0.19	0.061 *	−0.06	0.542
Apples and pears	−0.14	0.176	−0.23	0.022 **	−0.04	0.693
Avocado	0.09	0.365	0.01	0.960	0.25	0.014 **
Olives	0.05	0.613	0.02	0.839	0.09	0.409
Dried fruits	0.01	0.917	−0.06	0.566	0.18	0.083 *
Sweet fruit preserves and candied fruits	−0.13	0.196	−0.13	0.196	−0.02	0.843
Vegetables—all types	0.01	0.964	−0.05	0.605	0.16	0.125
Crucifers	0.08	0.429	0.05	0.620	0.02	0.869
Yellow–orange vegetables	−0.03	0.779	−0.11	0.264	0.12	0.235
Green leafy vegetables	0.11	0.273	0.09	0.393	0.09	0.368
Tomatoes	−0.06	0.537	−0.05	0.601	0.04	0.680
Vegetables: fresh cucumbers, squash, zucchini, pumpkin, eggplant	−0.12	0.238	−0.14	0.181	−0.10	0.338
Root vegetables and others	0.13	0.188	0.12	0.234	0.06	0.587
Fresh seeds of legumes and canned ones	0.00	0.966	−0.03	0.799	0.10	0.311
Dry seeds of legumes	−0.03	0.755	0.00	0.984	0.09	0.364
Potatoes in various forms	−0.06	0.578	−0.04	0.703	−0.13	0.203
Cereal products						
Wholemeal or with grains, so-called dark bread	−0.08	0.432	−0.14	0.163	0.16	0.124
Refined bread, so-called white bread	−0.26	0.010 **	−0.17	0.097 *	−0.24	0.019 **
Unrefined groats coarse	0.04	0.696	−0.05	0.612	0.12	0.234
Refined cereal grain	0.01	0.888	−0.04	0.673	0.22	0.030 **
Ready-to-eat breakfast cereal products	−0.11	0.264	−0.13	0.191	0.12	0.225
Dairy products and eggs						
Milk and milk drinks	−0.03	0.807	−0.04	0.682	0.10	0.354
Sweetened milk drinks	−0.13	0.182	−0.09	0.371	−0.08	0.416
Cottage cheese	−0.09	0.394	−0.04	0.717	−0.09	0.368
Flavored cottage cheese	−0.12	0.235	−0.13	0.195	−0.05	0.603
Cheese	−0.14	0.166	−0.10	0.323	−0.11	0.281
Eggs and egg dishes	0.06	0.585	−0.06	0.539	0.29	0.004 **
Meat products and fish						
Sausages, different types	−0.31	0.001 **	−0.20	0.052 *	−0.35	<0.001 **
High-quality cold cuts	−0.30	0.002 **	−0.12	0.219	−0.25	0.013 **
Sausage products and offal	−0.02	0.852	0.07	0.511	−0.18	0.074 *
Red meat	0.10	0.354	0.08	0.465	0.06	0.573
Poultry and rabbit	0.06	0.545	0.09	0.400	0.09	0.389
Wild game meat	−0.18	0.075 *	−0.13	0.212	−0.14	0.180
Lean fish	−0.01	0.913	−0.04	0.682	0.04	0.697
Oily fish	−0.03	0.764	−0.02	0.868	−0.04	0.722
Fats, nuts, and grains						
Oil, all kinds	−0.03	0.766	−0.13	0.194	0.05	0.614
Butter, all types	−0.20	0.043 **	−0.08	0.409	−0.38	<0.001 **
Margarine, all types	−0.23	0.020 **	−0.15	0.124	−0.19	0.065 *
Cream, sweet or sour cream, for food or beverages	−0.03	0.779	−0.01	0.940	−0.06	0.568
Other animal fats	−0.18	0.080 *	−0.13	0.206	−0.10	0.350
Mayonnaise and dressings, i.e., salad dressings—all types	−0.14	0.178	−0.04	0.676	−0.32	0.001 **
Nuts	0.11	0.273	0.08	0.444	0.13	0.202
Grains	0.10	0.313	−0.04	0.669	0.15	0.153
Sweets and snacks						
Sugar to sweeten beverages	−0.11	0.290	−0.01	0.895	−0.31	0.002 **
Honey to sweeten food and beverages	−0.10	0.348	−0.18	0.070 *	0.02	0.851
Chocolate, chocolate candies, and candy bars	−0.07	0.486	−0.04	0.687	−0.09	0.386
Non-chocolate candies	0.03	0.789	0.03	0.738	−0.06	0.570
Biscuits and cakes	−0.17	0.103	−0.10	0.327	−0.23	0.025 **
Ice cream and pudding	0.00	0.990	−0.05	0.595	−0.06	0.585
Salty snacks	−0.08	0.460	−0.02	0.853	−0.23	0.022 **
Soft drinks						
Fruit juices and fruit nectars	−0.31	0.001 **	−0.20	0.050 *	−0.28	0.004 **
Vegetable juices and vegetable-fruit ones	0.03	0.750	0.01	0.886	0.08	0.442
Energy drinks	−0.16	0.104	−0.13	0.188	−0.12	0.251
Sweetened sodas such as Fanta, Coca-Cola, Mirinda, Sprite	−0.24	0.017 **	−0.12	0.237	−0.34	<0.001 **
Alcoholic beverages						
Beer	0.05	0.593	0.20	0.050 *	−0.15	0.146
Wine and drinks	0.03	0.768	0.01	0.908	0.01	0.920
Vodka and spirits	0.20	0.044 **	0.26	0.009 **	−0.12	0.231

Spearman’s correlation; * statistical tendency 0.05 ≤ *p* ≤ 0.1; ** statistical significance *p* < 0.05.

**Table 6:** Relationship between physical activity and body mass index (BMI), fat mass index (FMI), and bone mineral density expressed as T-score.

Physical Activity	BMI		FMI		BMD T-score	
	rho	*p*	rho	*p*	rho	*p*
Physical activity (MET-minutes/week):						
total	0.07	0.518	−0.22	0.036	0.28	0.008 **
job-related physical activity	−0.01	0.934	−0.13	0.233	0.20	0.063 *
transportation	0.02	0.883	0.05	0.659	0.17	0.099 *
housework, house maintenance and caring for family	0.05	0.667	0.04	0.701	0.22	0.040 **
recreation, sport, and leisure-time	0.03	0.784	−0.13	0.194	0.25	0.019 **
walking	0.09	0.378	−0.01	0.900	0.20	0.062 *
moderate	0.03	0.762	0.06	0.585	0.28	0.008 **
intensive	−0.04	0.697	−0.22	0.036 **	0.21	0.046 **
Time spent sitting (h/d):						
weekdays	0.14	0.187	0.18	0.092 *	0.04	0.741
weekend	0.13	0.247	0.21	0.053 *	−0.07	0.530

Spearman’s correlation; * statistical tendency 0.05 ≤ *p* ≤ 0.1; ** statistical significance *p* < 0.05

## Supplementary Materials

The following are available online at https://www.mdpi.com/2072-6643/12/1/242/s1, Table S1. Comparison of food frequency consumption between soldiers with adequate and inadequate nutritional status (BMI, FMI, BMD T-score).

## References

[1] World Health Organization (2010). Global Status Report on Noncommunicable Diseases. http://apps.who.int/iris/bitstream/10665/44579/1/9789240686458_eng.pdf.

[2] Hupin D., Edouard P., Gremeaux V., Garet M., Celle S., Pichot V., Maudoux D., Barthélémy J., Roche F. (2017). Physical activity to reduce mortality risk. Eur. Heart J..

[3] Lee I., Skerett P.J. (2001). Physical activity and all-cause mortality: What is the dose-response relation?. Med. Sci. Sports Exerc..

[4] Blair S. (2009). Physical inactivity: The biggest public health problem of the 21st century. Br. J. Sports Med..

[5] Smith T.J., Marriott B.P., Dotson L., Bathalan G.P., Funderburk L., White A., Hadden L., Young A.J. (2012). Overweight and obesity in military personnel: Sociodemographic predictors. Obes. Res. J..

[6] Reyes-Guzman C.M., Bray R.M., Forman-Hoffman V.L., Williams J. (2015). Overweight and Obesity Trends Among Active Duty Military Personnel. A 13-Year Perspective. Am. J. Prev. Med..

[7] Clark H.L., Heileson J., DeMay J., Cole R.E. (2017). Misperceptions of Weight Status in Military Men and Women. Mil. Med..

[8] Kyoung-Ki B., Ho K., Sung-Il C. (2011). Trends in body mass index and associations with physical activity among career soldiers in South Korea. J. Prev. Med. Public Health.

[9] Sedek R., Koon P.B., Noor I.M. (2010). Body mass index and body composition among Royal Malaysian Navy (RMN) Personnel. J. Def. Secur..

[10] Lieberman H.R., Bathalton G.P., Falco C.M., Morgan C.A., Niro P.J., Tharion W.J. (2005). The fog of war: Decrements in cognitive performance and mood associated with combat-like stress. Aviat. Space Env. Med..

[11] Tomczak A., Kalina R.M., Sokołowski M. (2007). Appraisal of soldiers’ acquired skills for surviving in conditions of isolation. Morphofunctional Aspects of Selection of Soldiers for Realization of Tasks in the Army Formations.

[12] Crawford K., Fleishman K., Abt J.P., Sell T.C., Lovalekar M., Nagai T., Deluzio J., Rowe R.S., McGrail M.A., Lephart S.M. (2011). Less body fat improves physical and physiological performance in army soldiers. Mil. Med..

[13] Atlantis E., Martin S.A., Haren M.T., Taylor A.W., Wittert G.A., Florey Adelaide Male Aging Study (2008). Lifestyle factors associated with age-related differences in body composition: The Florey Adelaide Male Aging Study. Am. J. Clin. Nutr..

[14] Larson D., Hunter G., Williams M., Kekes-Szabo T., Nyikos I., Goran M. (1996). Dietary fat in relation to body fat and intraabdominal adipose tissue: A crosssectional analysis. Am. J. Clin. Nutr..

[15] Koppes L.L., Boon N., Nooyens A.C., van Mechelen W., Saris W.H. (2009). Macronutrient distribution over a period of 23 years in relation to energy intake and body fatness. Br. J. Nutr..

[16] Soenen S., Westerterp Plantenga M.S. (2010). Changes in body fat percentage during body weight stable conditions of increased daily protein intake vs. control. Physiol. Behav..

[17] Vinknes K.J., de Vogel S., Elshorbagy A.K., Nurk E., Drevon C.A., Gjesdal C.G., Tell G.S., Vollset S.E., Refsum H. (2011). Dietary intake of protein is positively associated with percent body fat in middle-aged and older adults. J. Nutr..

[18] Bowen L., Taylor A.E., Sullivan R., Ebrahim S., Kinra S., Krishna K.V., Kulkarni B., Ben-Shlomo Y., Ekelund U., Wells J.C. (2015). Associations between diet, physical activity and body fat distribution: A cross sectional study in an Indian population. BMC Public Health.

[19] Jarosz M., Wolnicka K., Sajór W., Wierzejska R., Jarosz M. (2017). Zasady dotyczące żywienia I aktywności fizycznej (Principles of nutrition and physical activity). Normy Żywienia dla Populacji Polski (Nutrition Norms for the Polish Population).

[20] Wądołowska L. (2005). Walidacja kwestionariusza częstotliwości spożycia żywności—FFQ. Ocena powtarzalności (Validation of food frequency questionnaire—FFQ. Reproducibility assessment). Bromat. Chem. Toksykol..

[21] International Physical Activity Questionnaire—Long Form, Polish Version. https://sites.google.com/site/theipaq/questionnaire_links.

[22] (2005). Guidelines for Data Processing and Analysis of the International Physical Activity Questionnaire (IPAQ). https://sites.google.com/site/theipaq/scoring-protocol.

[23] WHO (2000). Obesity: Preventing and Managing the Global Epidemic. Report of a WHO Consultation.

[24] Kelly T., Wilson W., Heymsfield B. (2009). Dual Energy X-Ray Absorptiometry Body Composition Reference Values from NHANES. PLoS ONE.

[25] Nojiri S., Burge R.T., Flynn J.A., Foster S.A., Sowa H. (2013). WHO Scientific Group on the Assessment of Osteoporosis at Primary Health Care Level: Summary Meeting Report, Brussels, Belgium, 5–7 May 2004. J. Bone Miner. Metab..

[26] Hyżyk A.K., Krejpcio Z., Dyba S. (2011). Ocena sposobu żywienia żołnierzy w wybranych jednostkach wojskowych (Evaluation of the method of soldiers’ feeding in selected army units). Probl. Hig. Epidemiol..

[27] Farina E.K., Taylor J.C., Means G.E., Murphy N.E., Pasiakos S.M., Lieberman H.R., McClung J.P. (2017). Effects of deployment on diet quality and nutritional status markers of elite U.S. Army special operations forces soldiers. Nutr. J..

[28] Piątkowska M. (2012). Self-rated physical activity across Europe—Poland and other European countries. Biol. Sport.

[29] Sjöström M., Oja P., Hagströmer M., Smith B.J., Bauman A. (2006). Health-enhancing physical activity across European Union countries: The Eurobarometer study. J. Public Health.

[30] Tomczak A. (2012). Physical activity of soldiers in the Polish Armed Force’s military administration units and special units. Biomed. Hum. Kinet..

[31] Correa-Rodríguez M., Rueda-Medina B., González-Jiménez E., Schmidt-RioValle J. (2017). Associations between body composition, nutrition, and physical activity in young adults. Am. J. Hum. Biol..

[32] Nuttall F. (2015). Body Mass Index. Obesity, BMI, and Health: A Critical Review. Nutr. Today.

[33] Meeuwsen S., Horgan G.W., Elia M. (2010). The relationship between BMI and percent body fat, measured by bioelectrical impedance, in a large adult sample is curvilinear and influenced by age and sex. Clin. Nutr..

[34] Romero-Corral A., Somers V.K., Sierra-Johnson J., Thomas R.J., Collazo-Clavell M.L., Korinek J., Allison T.G., Batsis J.A., Sert-Kuniyoshi F.H., Lopez-Jimenez F. (2008). Accuracy of body mass index in diagnosing obesity in the adult general population. Int. J. Obes..

[35] Prentice A.M., Jebb S.A. (2001). Beyond body mass index. Obes. Rev..

[36] Mullie P., Vansant G., Hulens M., Clarys P., Degrave E. (2008). Evaluation of body fat estimated from body mass index and impedance in Belgian male military candidates: Comparing two methods for estimating body composition. Mil. Med..

[37] Ode J., Pivarnki J., Reeves M., Knous J. (2007). Body mass index as a predictor of percent fat in college athletes and nonathletes. Med. Sci. Sports Exerc..

[38] Witt K., Bush E. (2005). College athletes with an elevated body mass index often have a high upper arm muscle area, but not elevated triceps and subscapular skinfolds. J. Am. Diet. Assoc..

[39] Grier T., Canham-Chervak M., Sharp M., Jones B.H. (2015). Does body mass index misclassify physically active young men. Prev. Med. Rep..

[40] Bertrandt J., Kłos A., Łakomy R., Maculewicz E. (2018). Stan odżywienia i uwapnienia kości żołnierzy 16 Batalionu Powietrzno-Desantowego (Nutritional status and bone calcification of soldiers serving in 16th Airborne Battalion). Probl. Hig. Epidemiol..

[41] Barringer N.D., Pasiakos S.M., McClung H.L., Crombie A.P., Margolis L.M. (2018). Prediction equation for estimating total daily energy requirements of special operations personnel. J. Int. Soc. Sports Nutr..

[42] Nour M., Lutze S.A., Grech A., Allman-Farinelli M. (2018). The Relationship between Vegetable Intake and Weight Outcomes: A Systematic Review of Cohort Studies. Nutrients.

[43] Moore L.V., Hamner H.C., Kim S.A., Dalenius K. (2016). Common ways Americans are incorporating fruits and vegetables into their diet: Intake patterns by meal, source and form, National Health and Nutrition Examination Survey 2007–2010. Public Health Nutr..

[44] Sharma S.P., Chung H.J., Kim H.J., Hong S.T. (2016). Paradoxical Effects of Fruit on Obesity. Nutrients.

[45] Van Eekelen E., Beulens J.W.J., Geelen A., Schrauwen-Hinderling V.B., Lamb H., de Roos A., Rosendaal F., de Mutsert R. (2019). Consumption of Alcoholic and Sugar-Sweetened Beverages is Associated with Increased Liver Fat Content in Middle-Aged Men and Women. J. Nutr..

[46] Prentice A. (2004). Diet, nutrition and the prevention of osteoporosis. Public Health Nutr..

[47] Wang P., Zhang H. (2003). Review of dietary risk factors for osteoporosis. J. Hyg. Res..

[48] Cohen A.J., Roe F.J. (2000). Review of risk factors for osteoporosis with particular reference to a possible aetiological role of dietary salt. Food Chem. Toxicol..

[49] Lupsa B.C., Insogna K. (2015). Bone Health and Osteoporosis. Endocrinol. Metab. Clin. N. Am..

[50] Bertrandt J., Kłos A., Łakomy R., Kaiser A., Mrozowiak M. (2013). Ocena uwapnienia kości żołnierzy polskich powracających z misji pełnionej w ramach Międzynarodowych Sił Wsparcia Bezpieczeństwa (ISAF) w Afganistanie (Evaluation of bone calcification of Polish soldiers returning from a mission performed as part of the International Security Assistance Force (ISAF) in Afghanistan). Zdrowotne i Psychospołeczne Aspekty Służb Mundurowych (Health and Psychological Issues of Uniformed Services).

[51] Langsetmo L., Hitchcock C.L., Kingwell E.J., Davison K.S., Berger C., Forsmo S., Zhou W., Kreiger N., Prior J.C. (2012). Physical activity, body mass index and bone mineral density-associations in a prospective population-based cohort of women and men: The Canadian Multicentre Osteoporosis Study (CaMos). Bone.

[52] Alghadir A.H., Gabr S.A., Al-Eisa E. (2015). Physical activity and lifestyle effects on bone mineral density among young adults: Sociodemographic and biochemical analysis. J. Phys. Sci..

[53] Correa-Rodríguez M., Rio-Valle J.S., González-Jiménez E., Rueda-Medina B. (2016). The Effects of Body Composition, Dietary Intake, and Physical Activity on Calcaneus Quantitative Ultrasound in Spanish Young Adults. Biol. Res. Nurs..

[54] Tucker K.L., Troy L., Morita K., Cupples A.L., Hannan M.T., Kiel D.P. (2003). Carbonated beverages consumption and bone mineral density. Am. Soc. Bone Miner. Res..

[55] Calvo M.S., Tucker K.L. (2013). Is phosphorus intake that exceeds dietary requirements a risk factor in bone health?. Ann. N. Y. Acad. Sci..

[56] Bertrandt J., Kłos S., Szymańska W., Walecka I. (2011). Wpływ specyfiki studiów na stan odżywienia białkowo-energetycznego i mineralnego studentów i roku Szkoły Głównej Służby Pożarniczej (SGPS) (An effect of the study specificity on protein-energy and mineral nutritional status of the Main School of Fire Service (MSFS) students). Pol. Przegląd Med. I Psychol. Lotniczej.

[57] Hauger A.V., Bergland A., Holvik K., Ståhle A., Emaus N., Strand B.H. (2018). Osteoporosis and osteopenia in the distal forearm predict all-cause mortality independent of grip strength: 22-year follow-up in the population-based Tromsø Study. Osteoporos Int..

[58] Choksi P., Jepsen K., Clines G.A. (2018). The challenges of diagnosing osteoporosis and the limitations of currently available tools. Clin. Diabetes Endocrinol..

