# Supplementary material for: Association Between Diet, Physical Activity and Body Mass Index, Fat Mass Index and Bone Mineral Density of Soldiers of the Polish Air Cavalry Units
Source: Nutrients. 2020 Jan 17;12(1):242. doi: 10.3390/nu12010242 (PMC7019523; doi:10.3390/nu12010242)
Supplement: Supplementary file 1 [file nutrients-12-00242-s001.pdf]

Table S1. Comparison of food frequency consumption between soldiers with adequate and inadequate nutritional status (BMI, FMI, BMD T-score).

Groups of foods	Adequate BMI		Adequate FMI		Adequate BMD T-score		p		
	YES	NO	YES	NO	YES	NO	BMI	FMI	BMD T-score
**Food groups**									
Fruits. vegetables, seeds of legume, and potatoes	5.96	5.52	5.59	5.96	5.68	5.12	0.582	0.643	0.859
Cereal products	2.22	1.74	1.84	2.15	1.90	2.37	0.007**	0.183	0.123
Dairy products and eggs	2.42	2.04	2.17	2.25	2.24	1.78	0.068*	0.549	0.284
Meat products and fish	2.55	1.92	2.05	2.44	2.11	2.99	0.009**	0.193	0.364
Fats, nuts, and grains	2.24	1.69	1.74	2.28	1.85	2.75	0.033**	0.012**	0.087*
Sweets and snacks	1.70	1.23	1.40	1.47	1.35	2.12	0.105	0.421	0.262
Non-alcoholic beverages	1.41	0.80	0.97	1.22	1.03	1.42	<0.001**	0.158	0.123
Alcoholic beverages	0.32	0.34	0.32	0.35	0.32	0.38	0.941	0.918	0.871
**Selected foods**									
Fruits, vegetables, seeds of legumes, and potatoes									
Fruits together—all types	0.74	0.65	0.69	0.67	0.67	0.62	0.790	0.475	0.988
Stone fruits	0.19	0.24	0.23	0.20	0.22	0.12	0.662	0.751	0.183
Kiwi fruit and citrus	0.21	0.25	0.23	0.23	0.24	0.18	0.813	0.687	0.285
Other tropical fruits	0.08	0.16	0.13	0.12	0.13	0.02	0.201	0.215	0.034**
Bananas	0.57	0.42	0.48	0.49	0.48	0.41	0.044**	0.775	0.858
Apples and pears	0.55	0.38	0.45	0.44	0.44	0.51	0.038**	0.966	0.513
Avocado	0.03	0.06	0.04	0.06	0.05	0.01	0.061*	0.111	0.124
Olives	0.05	0.10	0.07	0.10	0.09	0.01	0.152	0.085*	0.278
Dried fruits	0.15	0.08	0.13	0.07	0.12	0.02	0.827	0.091*	0.099*
Sweet fruit preserves and candied fruits	0.13	0.06	0.09	0.10	0.09	0.09	0.023**	0.783	0.287
Vegetables—all types	0.63	0.62	0.64	0.61	0.62	0.62	0.804	0.904	0.837
Crucifers	0.26	0.27	0.24	0.31	0.26	0.27	0.736	0.289	0.870
Yellow–orange vegetables	0.33	0.31	0.28	0.41	0.32	0.27	0.257	0.123	0.520
Green leafy vegetables	0.19	0.20	0.19	0.21	0.20	0.13	0.489	0.569	0.358
Tomatoes	0.54	0.44	0.46	0.52	0.47	0.44	0.248	0.401	0.621
Vegetables: fresh cucumbers, squash, zucchini, pumpkin, eggplant	0.43	0.36	0.37	0.43	0.38	0.38	0.237	0.564	0.973
Root vegetables and others	0.30	0.36	0.34	0.31	0.34	0.27	0.693	0.541	0.500
Fresh seeds of legumes and canned ones	0.13	0.13	0.14	0.12	0.13	0.13	0.726	0.385	0.758
Dry seeds of legumes	0.08	0.08	0.08	0.09	0.08	0.11	0.989	0.858	0.952
Potatoes in various forms	0.46	0.41	0.40	0.51	0.43	0.51	0.422	0.138	0.429

**Cereal products**									
Wholemeal or with grains, so-called dark bread	0.61	0.52	0.56	0.55	0.55	0.55	0.432	0.957	0.789
Refined bread, so-called white bread	0.76	0.41	0.50	0.66	0.51	1.05	<0.001**	0.044**	0.055*
Unrefined groats coarse	0.25	0.27	0.25	0.29	0.27	0.22	0.636	0.700	0.691
Refined cereal grain	0.34	0.32	0.33	0.33	0.32	0.35	0.900	0.894	0.724
Ready-to-eat breakfast cereal products	0.28	0.22	0.21	0.34	0.26	0.20	0.155	0.494	0.730
**Dairy products and eggs**									
Milk and milk drinks	0.54	0.55	0.57	0.50	0.56	0.33	0.695	0.854	0.200
Sweetened milk drinks	0.36	0.23	0.27	0.33	0.30	0.14	0.031**	0.183	0.257
Cottage cheese	0.42	0.35	0.38	0.37	0.38	0.38	0.142	0.208	0.946
Flavored cottage cheese	0.15	0.10	0.11	0.13	0.11	0.16	0.102	0.379	0.733
Cheese	0.48	0.32	0.33	0.50	0.38	0.50	0.051*	0.140	0.249
Eggs and egg dishes	0.46	0.50	0.52	0.42	0.50	0.27	0.826	0.257	0.042**
**Meat products and fish**									
Sausages, different types	0.71	0.34	0.45	0.59	0.46	0.93	<0.001**	0.117	0.230
High-quality cold cuts	0.63	0.41	0.46	0.58	0.47	0.78	0.003**	0.158	0.182
Sausage products and offal	0.15	0.15	0.14	0.16	0.15	0.19	0.621	0.110	0.894
Red meat	0.26	0.31	0.29	0.27	0.29	0.16	0.375	0.959	0.256
Poultry and rabbit	0.42	0.48	0.45	0.49	0.46	0.38	0.486	0.414	0.569
Wild game meat	0.05	0.02	0.05	0.01	0.04	0.02	0.023**	0.072*	0.619
Lean fish	0.17	0.12	0.13	0.16	0.12	0.29	0.991	0.780	0.676
Oily fish	0.18	0.09	0.10	0.18	0.11	0.28	0.416	0.594	0.582
**Fats, nuts and grains**									
Oil, all kinds	0.38	0.34	0.34	0.39	0.36	0.39	0.349	0.380	0.654
Butter, all types	0.71	0.50	0.54	0.70	0.55	1.02	0.021**	0.242	0.006**
Margarine, all types	0.39	0.15	0.19	0.36	0.23	0.47	0.008**	0.149	0.178
Cream, sweet or sour cream. for food or beverages	0.15	0.19	0.13	0.26	0.18	0.12	0.531	0.137	0.585
Other animal fats	0.09	0.05	0.06	0.09	0.06	0.17	0.338	0.991	0.151
Mayonnaise and dressings, i..e. salad dressings—all types	0.24	0.14	0.18	0.18	0.17	0.37	0.073*	0.169	0.055*
Nuts	0.20	0.20	0.20	0.20	0.21	0.11	0.255	0.641	0.098*
Grains	0.09	0.12	0.10	0.13	0.11	0.09	0.654	0.689	0.323
**Sweets and snacks**									
Sugar to sweeten beverages	0.71	0.52	0.54	0.72	0.55	1.02	0.098*	0.155	0.025**
Honey to sweeten food and beverages	0.20	0.18	0.21	0.14	0.17	0.28	0.691	0.007**	0.779

Chocolate, chocolate candies, and candy bars	0.33	0.22	0.27	0.25	0.25	0.44	0.386	0.751	0.642
Non-chocolate candies	0.12	0.08	0.10	0.09	0.10	0.04	0.976	0.314	0.664
Biscuits and cakes	0.17	0.13	0.15	0.14	0.14	0.25	0.083*	0.409	0.371
Ice cream and pudding	0.05	0.04	0.04	0.05	0.04	0.03	0.922	0.918	0.569
Salty snacks	0.14	0.09	0.12	0.08	0.12	0.05	0.280	0.394	0.676
Soft drinks									
Fruit juices and fruit nectars	0.64	0.33	0.43	0.52	0.43	0.74	<0.001**	0.424	0.095*
Vegetable juices and vegetable-fruit ones	0.18	0.15	0.17	0.16	0.18	0.06	0.451	0.970	0.176
Energy drinks	0.24	0.12	0.17	0.18	0.18	0.18	0.051*	0.855	0.868
Sweetened sodas such as Fanta. Coca-Cola, Mirinda, Sprite	0.35	0.20	0.21	0.36	0.25	0.45	0.005**	0.015**	0.050*
Alcoholic beverages									
Beer	0.22	0.18	0.20	0.19	0.19	0.31	0.430	0.705	0.515
Wine and drinks	0.05	0.08	0.06	0.08	0.07	0.04	0.994	0.803	0.556
Vodka and spirits	0.05	0.08	0.06	0.08	0.07	0.03	0.261	0.381	0.180

U Mann-Whitney Test

* statistical tendency $0.05 \leq p \leq 0.1$

** statistical significance $p < 0.05$
